# Factors influencing student’s transition from student to workforce in intensive care units

**DOI:** 10.4102/curationis.v48i1.2718

**Published:** 2025-04-30

**Authors:** Leonie Olivier, Charlene Downing

**Affiliations:** 1 Department of Nursing, Faculty of Health Science, University of Johannesburg, Johannesburg, South Africa

**Keywords:** practice readiness, nursing student, intensive care unit, novice general nurse, transition program, clinical learning environment

## Abstract

**Background:**

Staff shortages, the coronavirus disease 2019 pandemic, increased patient acuity and fiscal restraints contributed to basic nursing students being assigned to intensive care units (ICU). These students, expected to step up and function within the team, were confronted with expectations and situations beyond their clinical preparation for practice. How can we better prepare these students for practice?

**Objectives:**

To develop recommendations that promote transition programmes that prepare student nurses to become practice-ready novice general nurses to work in the ICU.

**Method:**

The study adopted a sequential explanatory mixed-method. Quantitative data collection was achieved through census sampling and the utilisation of the Casey Fink Practice Readiness Survey. Statistical analyses used IBM SPSS (version 25, IBM Corporation) to identify predictive relationships between practice readiness and identified variables through multi-linear regression. Qualitative data collected through purposive selection and semi-structured focus group discussions were transcribed, coded and analysed through domain analysis.

**Results:**

Four factors affecting nursing students’ perceived readiness for practice in the ICU were identified: (1) Support for new general nurses, (2) their need for professional socialisation and belonging, (3) orientation and skill development and (4) rotation and exposure to the ICU.

**Conclusion:**

Multifaceted innovative introduction programmes may assist in preparing the novice general nurse to become practice-ready.

**Contribution:**

This article contributes towards a possible solution to bridge the theory-practice gap and positively influence students’ transition into the workplace to facilitate retention of novice practitioners beyond their first year of practice in a specialised unit.

## Introduction

Nurse shortages related to the ageing nursing population, insufficient training opportunities and the inability to retain qualified nursing staff in specialised units is an international concern (Hampton, Smeltzer & Ross 2021; Hawkins, Jeong & Smith 2019; Splitgerber, Davies & Laker 2021). The magnitude of this problem was once again highlighted during the fight against the coronavirus disease 2019 (COVID-19) pandemic in 2020. The placement of final-year diploma nursing students into the intensive care units (ICU) became fundamental to attaining the required nursing capacity to provide for the sudden increase in patients with high acuity (Hampton et al. 2021; Hawkins et al. 2019; Splitgerber et al. 2021). These students were subjected to situations far beyond their basic nursing preparation (De Swardt 2019; Drennan & Ross 2019; Hawkins et al. 2019; Kaihlanen et al. 2020; Wiredu & Roberts 2020). The expectation of commencing their tasks immediately and proficiently became their reality, provoking fear, anxiety and feelings of incompetence because of their lack of experience, inability to integrate theory with practice, inadequate support structures and disregard for policy and procedure (De Swardt 2019; Drennan & Ross 2019; Hawkins, et al. 2019; Kaihlanen et al. 2020; Wiredu & Roberts 2020). The lack of orientation and professional socialisation exacerbated this situation leaving many feeling destitute and alienated.

### Background within the South African context

Acquisition of the skills and values to become a practice-ready novice general nurse with the ability to take responsibility can only be obtained through experience-based knowledge. This knowledge is supported by structured facilitation and further enhanced through prolonged exposure to the clinical learning environment (CLE) (Hampton et al. 2021; Mirza et al. 2019; Murray, Sundin & Cope 2019; Rojo et al. 2020; Rush et al. 2019). South African basic nursing education programmes aim to develop the newly qualified nurse’s ability to accept responsibility and demonstrate accountability. This is achieved by encouraging self-awareness of limitations, exercising professional judgement and seeking consultation from qualified senior staff and role models (Hampton et al. 2021; Harrison et al., 2020; Innis & Calleja 2018; Rush et al. 2019).

### Integration of theory into practice

Through prolonged exposure and by encouraging students to participate in real-time learning opportunities, active integration of theory and practice can be achieved (Hampton et al. 2021; Hattingh 2019; Kaihlanen et al. 2020). A prerequisite for registering as a bridging course student is being registered with the South African Nursing Council (SANC) as an enrolled nurse (R683 of 1989). This requirement ensures that all the students in this study have obtained a minimum of 2000 h of exposure to the CLE through work-integrated learning (WIL) before their registration as bridging course students (*Nursing Education and Training Standards; Act 33 of 2005*). During the bridging course, the student’s clinical exposure and requirements as stipulated by the SANC (*Nursing Education and Training Standards; Act 33 of 2005*) included an additional 2000 WIL hours to enable them to register as a general nurse (R683 of 1989). Participants were placed within accredited private healthcare facilities that could facilitate rotation through all the required disciplines to meet the requirements of the SANC. Placement of students in high care or ICU is not a requirement stipulated by the SANC (*Nursing Education and Training Standards; Act 33 of 2005*). However, during the COVID pandemic, acuity constraints and a severe staff shortage contributed to the placements of final-year students in high care and ICU.

### Support structures

Many studies emphasise the importance of role models in the novice’s professional development (Comparcini et al. 2020; Hampton et al. 2021; Pleshkan & Hussey 2020). Moreover, peer support and the development of trust relationships assist the novices in asking for assistance from peers and seniors (Harrison et al. 2020; Menard & Maas 2019; Rush et al. 2019; Vuckovic, Karlsson & Sunnqvist 2019). The novice further needs extensive, objective support in the form of standard operating procedures, algorithms, guidelines and policy documents to guide clinical decision-making as they do not possess the required skills and critical thinking ability to act independently (Murray et al. 2019; Pitts & Christenbery 2019). In South Africa, clinical placements of students in the ICU are subject to the direct supervision of qualified staff (SA Nursing 2011). Unfortunately, the students who were placed as workforce in the ICU during the pandemic were not afforded structured and competent support from qualified senior staff. Instead, they were left to their own devices because of the severe restraints placed on the nursing fraternity.

### Professional socialisation and integration as a team member

It remains a national and international expectation that all senior staff members and shift leaders should act as preceptors and role models (Smith & Sweet 2019; Watkins, Hart & Mareno 2016). Positive experiences and the effect of professional role models to guide, support and embrace students into the ICU culture are powerful and empowering (Innis & Calleja 2018; Van Den Boogaard et al. 2019). Novice nurses who experience positive interactions within the clinical environment will thrive and feel safe enough to demonstrate their actual level of competence (Comparcini et al. 2020; Harrison et al. 2020; Vuckovic et al. 2019). The novice who feels safe and supported and experiences a sense of belonging is more likely to accept responsibility and accountability for patient care (Inis & Calleja 2018; Murray et al. 2019; Rush et al. 2019).

### Orientation

Orientation is one of the cornerstones to a successful transition into a new work environment (Pryse et al. 2020; Rush et al. 2019; Song & McCreary 2020). It is well documented that the transition into the ICU environment is more difficult because of the specialised knowledge and skills needed to provide quality patient care (Innis & Calleja 2018; Sterner et al. 2019; Williamson, Kane & Bunce 2020). Orientation should include unit routines, roles and responsibilities, available support structures and the development of cognitive competencies and skills required to reduce anxiety and fear of the unknown (Hampton et al. 2021; Harrison et al. 2020; Rush et al. 2019; Wiredu & Roberts 2020).

### Conceptual framework

This study is underpinned by Benner’s theory of novice to expert. By adopting the Dreyfus model, Benner described five levels of skill acquisition in chronological order: (1) novice, (2) advanced beginner, (3) competent, (4) proficient and (5) expert (Alligood 2014; Landers, O’Mahony & McCarthy 2020; Murray et al. 2019; Thomas & Kellgren 2017). Benner’s theory emphasises that newly qualified nurses allocated to work in the ICU are novice practitioners based on their experience and exposure (Alligood 2014; Benner 2001; Gobet & Chassy 2008; Landers et al. 2020; Murray et al. 2019;Stinson 2017; Thomas & Kellgren 2017). The novice relies on continuous support and guidance along with clear policies and procedures to govern their behaviour (Alligood 2014; Benner 2001; Gobet & Chassy 2008; Landers et al. 2020; Murray et al. 2019; Stinson 2017; Thomas & Kellgren 2017). These novice practitioners develop skills and understanding of patient care over time through the integration of sound theoretical knowledge and practical experiences (Alligood 2014; Benner 2001; Gobet & Chassy 2008; Landers et al. 2020; Murray et al. 2019; Stinson 2017; Thomas & Kellgren 2017). Benner further emphasises that the novice will progress to an advanced beginner as clinical exposure to situational learning experiences increases (Murray et al., 2019).

### Research questions and study aim

In a bid to develop recommendations regarding support structures needed to facilitate the transition from student to novice practitioner who is practice ready, it became imperative to answer the following questions: (1) *What is final-year nursing students’ self-reported perception of their readiness for practice in the ICU?* (2) *What factors are identified by final-year nursing students that influenced their perception of readiness for practice in the ICU?* (3) *What is the relationship between undergraduate nursing students’ exposure to the critical care environment and the effect thereof on their perceived readiness for practice?*

## Research methods and design

### Design and sample

This study adopted an explanatory, sequential, mixed-method approach with the collection of quantitative data, followed by a qualitative strategy aimed at verifying and explaining the quantitative results (Creswell & Creswell 2017; Gray, Grove & Sutherland 2017; Polit & Beck 2018).

Quantitative data were collected online using the Casey Fink Practice Readiness Survey (CFPRS) (Casey, Tsai & Fink 2011). All registered final-year bridging course (R683) nursing students at a private nursing education institution in South Africa were invited to participate in the study (population *N* = 412) through a census sampling method.

#### Data collection instrument

Permission to use and adapt the CFPRS for the South African context was received from its creators. The CFPRS underwent a pilot study to assess the comprehensibility of the vocabulary used, the time it took to complete the survey and the ease of understanding the instructions. The adapted CFPRS consisted of two main sections and covered the respondents’ self-reported demographic data and clinical practical experience. The demographic components were adapted to the South African context regarding qualifications, training programmes and clinical environments selected for student placement during their training. Language adaptations were made by changing ‘clinical instructor’ to’ clinical facilitator’ and ‘physician’ to ‘doctor’. Section A consisted of 21 closed structured questions and started with general demographic questions such as age and gender and progressed to more population-related specific questions. The demographic data were obtained with the application of a nominal level of measurement. Data regarding the independent variables related to the students’ clinical exposure, support and orientation in the intensive care environment during their final year of study were also assessed. Section 2 of the questionnaire directly measured the dependable variable of the respondents’ perception of their readiness for practice and enquired about respondents’ opportunities to practice skills more than once during simulation. During the data analysis, four independent variables (support structure general nurse, shifts per month overtime, attended up-skill training and orientation) were found to have significant predictive value in relation to readiness for practice scores.

#### Validity and reliability

The CFPRS has been validated based on construct validity, face validity, factor and confirmatory factor analysis and has been used extensively in various international studies (Baker & Alghamdi 2020; Jamieson et al. 2019). The reliability of the CFPRS instrument was already established and has a reported Cronbach’s alpha coefficient of 0.89 (Baker & Alghamdi 2020; Jamieson et al. 2019).

#### Semi-structured focus group discussions

The quantitative method was sequentially followed by a qualitative method and purposive sampling, through the application of two semi-structured focus group discussions consisting of four and five participants, respectively. The participants were purposively selected as they were respondents in the first phase of the study and had extensive experience of being expected to assist during the COVID-19 pandemic. Data saturation was reached during the second focus group (Gray et al. 2017; Polit & Beck 2018). The questions for the focus group discussions were derived from the results obtained during the quantitative phase. Questions asked addressed the four independent variables that influenced the student nurse’s perception of readiness for practice in a bid to gain a deeper understanding of how these factors influenced their perceived readiness for practice. These questions were: (1) Can you please explain to me or describe to me how would you describe your readiness for practice in the ICU; (2) Please can you explain or elaborate for me on what is your experience of the support received during your exposure as a student in the ICU environment; (3) What was your experience of the up-skill programme? And did you attend an up-skill programme or not; (4) How would you describe your orientation? and (5) Were there any other factors that you feel prepared you for practice readiness?

#### Credibility

Credibility was ensured through data triangulation, member checking, prolonged engagement and peer review (Gray et al. 2017; Polit & Beck, 2018).

#### Transferability

The transferability of the study’s findings was determined by the similarity of the situation in relation to the description of the purposive sampling method, a description of the participants’ demographic data, as well as the inclusion criteria for the selection of the sample population (Polit & Beck 2018).

#### Dependability

Dependability was ensured through a detailed description of the research design, methodology, sampling method, data collection and analysis method (Korstjens & Moser 2018).

#### Confirmability

Confirmability was ensured through the utilisation of a mixed-method research approach, data triangulation, member checking and the utilisation of an independent coder during qualitative data analysis (Polit & Beck 2018).

#### Trustworthiness

Trustworthiness was established as the researcher established credibility, dependability, transferability, authenticity and confirmability (Polit & Beck 2018).

#### Data collection

Data collection for this study was conducted from February 2021 to April 2022. Permission to be contacted by the researcher regarding the study was obtained by a gatekeeper. The link to the online information letter through the utilisation of Google Forms was provided to the student. Consent to be contacted was obtained from 186 students, and 109 students accessed the online survey. The response rate was 58.6%. This was followed by two semi-structured focus group discussions. The size of the focus groups was dependent on the number of participants who availed themselves of participation at the same time and logged on to attend the meeting on the day of the discussion. Four and five participants attended the focus groups, respectively. Data saturation was reached during the second focus group as no new data emerged (Gray et al. 2017; Polit & Beck 2018).

#### Data analyses

Data collected during the quantitative phase were captured using an online platform (Google Forms) and exported into an Excel spreadsheet. The data were cleaned and coded numerically (Boswell & Cannon 2020; Gray et al. 2017). IBM (SPSS) (version 27, IBM Corporation, New York, US) was used to analyse the data. Demographic and response variables were described by frequency analysis and calculation of means, 95% confidence intervals or medians and interquartile ranges. Differences in mean scores between groups assigned to specialised and peripheral hospitals during training were assessed using a *t*-test. Predictive relationships between readiness for practice scores, orientation, clinical exposure to the intensive care environment, support in the ICU and attendance of a skill development programme were assessed using multivariate linear regression.

During qualitative data analysis, domain analysis was performed by the researcher, supervisor and an independent coder. The verbatim transcriptions of the audio recordings of the focus group discussions were coded in the following manner: (1) Identification of primary domains, (2) constructing a taxonomy of sub-domains, (3) specifying the components and (4) the domains were related to each other. Meetings were conducted between the researcher, supervisor and independent coder to review and refine the identified domains and sub-domains. The final agreed-on themes, domains and sub-domains are summarised in Table 7. The integrated quantitative and qualitative results identified four factors that affected the nursing students’ perceived readiness for practice in the ICU environment: (1) Support for new graduates, (2) their need for professional socialisation and belonging, (3) orientation and skill development and (4) rotation and exposure in the clinical learning environment as a student. The results were supported by literature for integration purposes (Gray et al. 2017).

#### Ethical considerations

Ethical clearance to conduct this study was obtained from the University of Johannesburg Faculty of Health Sciences Research Ethics Committee (reference no.: REC-512-2020). Ethical approval was granted by the University of Johannesburg, the Private Nursing Education Institution and the affiliated Private Health Care Provider. Students consented to be contacted with regard to the study. Informed consent was obtained from the respondents for the quantitative as well as the qualitative phase of the study. Participation was thus voluntary, and confidentiality during the quantitative phase of the study was assured. Participants were made aware that complete confidentiality could not be assured during the qualitative phase of the study as the participants might know each other. However, the participants, the independent coder, as well as the moderator were asked to sign a confidentiality clause (Polit & Beck 2018). Consent to digital record the focus group sessions was also obtained.

## Results

### Quantitative results

As indicated in Table 1 only 88 responses could be utilised during quantitative data collection as not all the questionnaires were completed in full.

**TABLE 1:** Demographic and work characteristics.

Description	%	*n*
**Gender**		
Female	90.9	80
Male	9.1	8
**Age (years)**		
24–34	63.6	56
35–44	27.3	24
45–54	9.1	8
**Year registered for the final year of studies**		
June 2019	11.3	10
Jan 2020	30.7	27
Jan 2021	58.0	51
**Prior qualifications**		
Care worker	4.5	4
Axillary nurse	6.8	6
Pen 1	27.3	24
Pen 2	27.3	24
Enrolled nurse	100.0	88
**Experience gained by respondents as enrolled nurses before studies for the bridging course commenced**		
6 months	3.4	3
6–12 months	13.6	12
2 years	17.0	15
3 years	20.5	18
4 years	9.1	8
5 years	8.0	7
More than 5 years	28.4	25
**Clinical fields where the experience was gained prior to enrolment as a student (more than one option could be selected)**		
Surgical ward	18.1	52
Medical ward	17.4	50
Orthopaedic ward	10.1	29
Casualty	12.2	35
Adult ICU	10.8	31
Paediatric ICU	1.4	4
Neonatal ICU	4.2	12
Theatre and recovery	9.7	28
**Last unit of employment prior to enrolment as a student**		
Surgical ward	21.6	19
Medical ward	12.5	11
Orthopaedic ward	4.5	4
Casualty	10.2	9
Adult ICU	18.2	16
Paediatric ICU	1.1	1
Neonatal ICU	1.1	1
Theatre and recovery	4.5	1
Cardiac Ward	4.5	4
Paediatric ward	4.5	4
Unemployed	14.8	13
**Geographic distribution**		
Eastern Cape	2.3	2
Gauteng North	11.4	10
Gauteng South	63.5	56
KwaZulu-Natal	14.8	13
Western Cape	8.0	7

*Source:* Olivier, L., 2022, Practice readiness of final-year nursing students in intensive care in a private hospital group, Unpublished Masters dissertation, University of Johannesburg, Johannesburg

ICU, intensive care unit.

### Demographic data

#### Age and gender

The age distribution of respondents for this study ranged from 25 years to 53 years ([Mean] *M* = 32; standard deviation [s.d.] 7.032). The mean age of the target population was 33 years. Most respondents (63.6%; *n* = 58) were between the ages of 25 and 34. This age distribution is representative of the target population, with an average of 61% in the same age group (National Statistical Report of Private Education Institution 2021). Female respondents in this study accounted for 90.9% (*n* = 80) of the respondents, and 9.1% (*n* = 8) were male. The gender distribution for this study is representative of the target population, with 92.2% (*n* = 380) females and 7.8% (*n* = 32) males.

### Prior qualifications and related experience

Prior qualifications before enrolment as a bridging course student vary because of the nature of opportunities available to progress. Experience gained as well as the last unit of employment prior to enrolment to the bridging course as stipulated in Regulation 683 (R683) of the *Nursing Act* (*No. 33 of 2005*) varies because of personal circumstances and progression opportunities related to each individual student (Hampton et al. 2021:4). The geographic distribution of the respondents is representative of all five campuses nationally and in line with the size of the private education institution (PEI) in each province (National Statistical Report of Private Education Institution 2021.

#### Exposure to the intensive care unit clinical learning environment as a final-year nursing student

Work-integrated learning during the final year of studies varied based on the previous year’s clinical placements and planned rotation within available space in the CLE according to the requirements as stipulated by the SANC (*Nursing Education and Training Standards; Act 33 of 2005:4*). As indicated in Table 2, most respondents work voluntary overtime shifts in ICU and was compensated for these shifts. Respondents who worked more than four shifts per month accounted for 24% (*n* = 21). Respondents working four overtime shifts per month represented 10% (*n* = 9) of the sample, while 15% (*n* = 13) worked two overtime shifts. Respondents who worked three overtime shifts represented 5% (*n* = 4) of the sample, and the remaining 4% (*n* = 4) worked one overtime shift. The remaining 42.0% (*n* = 37) respondents did not work any voluntary overtime shifts in the ICU.

**TABLE 2:** Prolonged exposure to the clinical learning environment.

Description	%	*n*
**Period allocated to ICU CLE during final year (months):**		
Not allocated	25.0	22
< 1	14.0	12
1	21.0	18
2	16.0	14
3	13.0	11
4	1.0	1
5	2.0	2
6	2.0	2
> 6	6.0	6
**Frequency of voluntary compensated overtime shifts worked in the ICU:**		
No shifts	42.0	37
1	4.5	4
2	15.0	13
3	4.5	4
4	10.0	9
> 4	24.0	21

*Source:* Olivier, L., 2022, Practice readiness of final-year nursing students in intensive care in a private hospital group, Unpublished Masters dissertation, University of Johannesburg, Johannesburg

CLE, clinical learning environment; ICU, intensive care unit.

#### Orientation received as students allocated to the intensive care unit

As indicated in Table 3, 64.8% (*n* = 57) of respondents were orientated to the ICU environment, while 35.2% (*n* = 31) were not orientated. Most respondents (40.4%; *n* = 23) rated the quality of orientation as average, followed by 29.8% (*n* = 17) rating orientation quality as good.

**TABLE 3:** Orientation received during allocation to the intensive care unit clinical learning environment as a student.

Description	%	*n*
**Orientation received during the time allocated to the ICU as a student**		
No orientation received	35.2	31
Orientation received	64.8	57
**Quality of orientation**		
Very poor	7.0	4
Poor	3.5	2
Average	40.4	23
Good	29.8	17
Excellent	19.3	11

*Source:* Olivier, L., 2022, Practice readiness of final-year nursing students in intensive care in a private hospital group, Unpublished Masters dissertation, University of Johannesburg, Johannesburg

ICU, intensive care unit.

#### Support structures available to the students

As illustrated in Table 4, most respondents felt they were not supported by the CNS. They indicated that they were moderately supported by the unit manager, CF and other general nurses. The respondents indicated they were fully supported by the shift leader and their peers.

**TABLE 4:** Support structures available during allocation as a student to the intensive care unit clinical learning environment.

Description of staff	No support		Little support		Moderate support		A lot of support		Full support		No of responses	
	*n*	%	*n*	%	*n*	%	*n*	%	*n*	%	*n*	%
Unit manager	15	18.1	17	20.5	24	28.9	12	14.5	15	18.0	83	100.0
CF	19	23.8	12	15.0	23	28.8	13	16.2	13	16.2	80	100.0
CNS	24	33.8	14	19.7	15	21.1	8	11.2	10	14.2	71	100.0
Shift leader	3	3.6	16	19.3	26	31.3	17	20.5	21	25.3	83	100.0
Senior RN	4	4.9	8	9.8	28	34.1	22	26.8	20	24.4	82	100.0
Fellow student	6	7.7	10	12.8	9	24.4	20	25.6	23	29.5	68	100.0
EN	9	11.1	13	16.0	31	38.3	18	22.2	10	12.4	81	100.0
ENA	20	27.0	11	14.9	16	21.6	16	21.6	11	14.9	74	100.0

*Source:* Olivier, L., 2022, Practice readiness of final-year nursing students in intensive care in a private hospital group, Unpublished Masters dissertation, University of Johannesburg, Johannesburg

CNS, clinical nurse specialist; CF, clinical facilitator; RN, responsible nurse; EN, enrolled nurse; ENA, enrolled nursing assistant.

#### Opportunity to practice procedures during simulation

As indicated in Table 5, 78 of the respondents (92%) stated that they had the opportunity to practice skills in simulation during their basic training, and 67 respondents (77%) indicated they did not feel competent to perform these skills in the clinical environment yet. Respondents who indicated they felt simulation contributed to their readiness for practice accounted for 44% of the population.

**TABLE 5:** Opportunity to practice during simulation during up-skill training sessions and self-perceived readiness for practice as a novice in the intensive care unit.

Description of nursing skill	Total number of respondents		Strongly disagree (1)		Disagree (2)		Agree (3)		Strongly agree (4)		Mean score	Standard deviation
	*n*	%	*n*	%	*n*	%	*n*	%	*n*	%		
I feel ready for the professional nursing role in an ICU environment	87	100.0	26	29.9	41	47.2	15	17.2	5	5.7	1.99	0.842
Simulations have helped me feel prepared for practice in the ICU	86	100.0	11	12.8	27	31.4	40	46.5	8	9.3	2.52	1.836
I have had opportunities to practice skills and procedures more than once	85	100.0	4	4.7	3	3.5	41	48.3	37	43.5	3.31	0.756

*Source:* Olivier, L., 2022, Practice readiness of final-year nursing students in intensive care in a private hospital group, Unpublished Masters dissertation, University of Johannesburg, Johannesburg

ICU, intensive care unit.

#### Self-perceived readiness for practice

During an independent sample *t*-test, differences between mean readiness for practice scores for respondents allocated to peripheral (*n* = 16) versus specialist (*n* = 39) hospitals were compared as indicated in Table 6. The results illustrated that there was no significant difference between the two groups (mean difference = −0.270, 95% confidence interval for difference = −0.940; 0.401, *t* = −0.811, *p* = 0.422) with equal variances assumed (Levene’s test, *p* = 0.629).

**TABLE 6:** Mean readiness scores for respondents in different provinces allocated to peripheral and specialised hospitals for clinical experience.

Group	Province	*n*	Mean (s.d.)
Peripheral (*n* = 16)	Gauteng North	3	1.33
	Gauteng South	10	2.50
	KwaZulu-Natal	2	2.50
	Western Cape	1	-
	Eastern Cape	0	-
Specialised (*n* = 39)	Gauteng North	3	3.00
	Gauteng South	31	2.87
	KwaZulu-Natal	3	2.33
	Western Cape	2	2.00
	Eastern Cape	0	-

*Source:* Olivier, L., 2022, Practice readiness of final-year nursing students in intensive care in a private hospital group, Unpublished Masters dissertation, University of Johannesburg, Johannesburg

Note: Neither test identified a significant main effect of provinces on readiness for practice scores (peripheral group: *F* = 3.174, *p* = 0.064; specialised group: *F* = 0.713, *p* = 0.551).

s.d., standard deviation.

Two separate one-way ANOVA tests were conducted, comparing mean readiness scores across different provinces for respondents allocated to peripheral (*n* = 16) and specialised (*n* = 39) hospitals. There were no significant differences identified in different provinces on readiness for practice scores among respondents allocated to peripheral or specialised units (peripheral group: *F* = 3.174, *p* = 0.064; specialised group: *F* = 0.713, *p* = 0.551). These results are further supported by the results of the independent sample *t*-test, where the null hypothesis could not be ruled out.

### Qualitative results

During the qualitative data analysis, three domains and seven sub-domains were identified. The described domains and sub-domains are directly supported by participants’ quotes, as indicated in italics in Table 7.

**TABLE 7:** Summary of the domains and sub-domains related to the participants’ lived experiences that impacted their self-perceived readiness for practice in the intensive care unit.

Domain	Sub-domain
**Domain 1:** Participants experienced fear and anxiety when exposed to the reality of the ICU learning environment without proper orientation prior to working in the ICU	1.1. **Their fear of the unknown was related to:** The participants experienced fear of the unknown foreign reality of the ICU environment without proper orientation. ‘I never really had orientation to ICU. So Umm it was a bit, a bit difficult is was to be, to be actually in a new environment whereby you know almost nothing about, it was bad. So, Amm navigating the way around without being orientated, is you just ask your neighbour, or go to the shift leader and ask. If you’re fortunate you have somebody who’s gonne tell you things.’ (P9, F, 28 years old) Because of a lack of orientation on the routine, layout of the unit, and where to find the required stock and equipment, the participants felt lost, incompetent and overwhelmed. ‘Umm ok so from my experience I don’t wane lie, I won’t say that I was orientated in any ICU that I’ve worked in. It was, you walk in, there’s your, well you go to allocation you you’re your allocation you see you allocated bed one and two, you go there you don’t know what to do and in the end I also end up looking at the previous chart, how they did their nursing care plan and then I would do that. You end up looking for where’s the sluice room, where is the stock room, where’s all the equipment that you need. Cause for me I had to figure out everything. Where’s this where’s that.’ (P3, F, 27 years old) The participants, in their student capacity, were not being recognised as members of the team and felt unwelcome and isolated. ‘Some nurses will welcome you and umm make sure that you know what’s going on, but other nurses will totally ignore you and leave you to our own devices and that can be really, really overwhelming when you are still a student and a newly qualified general nurse.’ (P5, F, 24 years old) The participants feared the possibility of making a life-threatening mistake because of their lack of knowledge to operate the intimidating equipment. ‘Especially with not having prior amm practice the equipment so, you kinda like more worried that you amm going to accidentally press a button and then cause harm to the patient, or something that you would do wrong.’ (P2, M, 38 years old) 1.2. **Factors that contributed to the participants’ perceived preparedness for the ICU environment were described as follows:** The participants felt that proper orientation of at least 2 weeks on the environment, the equipment and the staff would have made them feel safe and confident. ‘I think at least two weeks of theory and aaa particles around [*slight pause*] the ICU environment the equipment amm the expectations excreta within your clinical settings, so for example you would have to go, and do all of that in your clinical department and then after the second week then you, you can be placed in an ICU to then aaa, put that practice in motion.’ (P2, M, 38 years old) The participants felt that the huge difference they experienced regarding orientation as a student compared to the agency staff influenced their perception of being ready to work in the ICU. ‘I think orientation umm in some ICU’s, especially if they know that no, this is a [*replacement of institution*] person, This person works here at [*replacement of hospital*], they know how to do things so they neglect to orientate us, but when an agency person comes from another hospital, [*slight pause*] they get more orientated than, I feel I was not orientated, cause for me I had to figure out everything.’ (P3, F, 27 years old) The participants described that rotation to the ICU as a student gave them the benefit of prior exposure, which reduced their anxiety and fear of the unknown. ‘So the difference was, when we were sent now to the ICU, it wasn’t our first time, first environment, were not scared any more, were not anxious to go to the ICU, cause we been expose, you gave us those days to be exposed and to the doctors in the ICU, exposed to the procedures that they do in the ICU it really did make the different on us and also you know you get used to the staff and also you not scared to see the ventilated patient.’ (P6, F, 38 years old) Participants felt that the lack of orientation led to boredom and a negative view of the ICU. ‘So ICU for me was very boring, and I didn’t know what to do, and everybody else was working, that was my first day in ICU when I was ahhhai this is, this is [*laugh*] this is, hi guys. I am even falling asleep, and even if I go ask them can I go help with anything, the only thing they will call me for is when they need to change nappies and bed bath. Nothing, that is like new and empowering me to learn more or ICU wise. like it’s a special unit, I want to learn more about the ventilator, how to suction and everything.’ (P8, F, 26 years old)
**Domain 2:** Participants shared the importance of being embraced into the ICU culture by role models and how it contributed to a sense of belonging and acceptance that allowed for significant in-the-moment learning.	2.1. **The participants described how the support of the shift leaders, enrolled nursing assistants, as well as their peers, enhanced their perceived readiness for practice in the ICU.** The role of the shift leaders was identified as the most important support structure that provides orientation and direct supervision for the students during their exposure to the ICU. ‘You know whenever you don’t understand, whenever you need help, she’s always [*disturbance in recording*] she’s got that heart of teaching other, other colleague, so she’s my she’s my role model, truly speaking because of her and she’s, she’s not only helping me she help everyone. I’m still happy working in [*replacement of unit*] because of her. I just hope like aa one day I will just aaa aa the, the hole I will be like her helping others [*disturbance in recording*] yes who’s starting in [*replacement of unit*] or other ICU [*Disturbance in recording*]. Shift leaders [*pause*] cos I think the aaa the other thing that I liked working in ICU is because you always have support [*pause*]. it’s not like in the wards, whatever you [*disturbance*] understand you have a fear in then you have a support, somebody to ask, shift leaders. Support has been there from day one. ICU was amazing [*disturbance*] you, you can never say it’s alright for you to do something wrong if you are able to ask, because whenever you ask, they are there for you to attend.’ (P4, F, 41 years old) The role of the ENA, as a support structure, is often overlooked. Because of their experience in the ICU, the ENAs could give the students excellent directions regarding the layout, routine and culture of the unit. ‘But it’s also for me the, the care workers the ENA’s within the ICU. They are also the once that [*pause*] can be able to give you an in-depth ahh ahhm tips on things that’s happening in the ICU because sometimes it’s about the whole routine in the ICU and [*pause*] with their guidance, or exposure and experience there, it also made it easier in terms of, the nitty gritty how to do etcetera.’ (P2, M, 38 years old) Some participants indicated that the support they received from their peers, and even those who were students before them, made them believe there is hope and that they too will be able to adjust to the ICU environment. ‘As a student’s you know each other, and it’s very nice I think a other student maybe they have people that have been forward before you at school. They are the people that support you a lot, more that the people they don’t know you. So as students even if we are done with BC we support each other. So, I think Umm that have been the biggest support that I have been getting. I don’t know about other people and some other aaa staff members, they were supportive, but I feel like people that I went to school with they were more supportive than people that don’t know me. I’ve worked in different ICU and then I saw that the other some, other people they take time to get to, to get used to you and support you. So, the more support that I received is from student that I went to school with. Some people they did the ICU course so and they were able to help me, and I would feel like I can go and ask them something than people like someone that I don’t know.’ (P7, F, 28 years old) Participants who experienced a lack of support explained that it made them feel unmotivated, with a negative attitude towards the ICU. ‘mm A I would say it depends on the ICU that you being allocated on, and it depends on the people in the ICU. Different ICU’s different people, different environments you can never experience love, support and Umm teamwork in every ICU. So, it depends on the kind of people that you get and the kind of ICU that you are being allocated to. The people do make the environment to be a bit difficult for us at times to adapt to.’ (P9, F, 28 years old) 2.2. **The participants experienced that optimal clinical learning experiences occur when:** The shift leader and permanent staff focussed on potential moments to teach the students, and the staff intentionally involved the students during learning opportunities. ‘The little experience that I have they taught me so much because we had so many resusses during this time. So, you gain so much of knowledge and experience, during that time. She’s the one who taught me lot of new things and who made me go ahead to be flexible and do it by myself, so she gave me that confidence.’ (P8, F, 26 years old)
**Domain 3:** The participants described that there is a need for the up-skill programme to equip and empower them to work independently with confidence.	3.1. **The participants described they would have benefited from the up-skill programme because:** The participants felt empowered by developing skills to integrate theory with knowledge and build confidence through skill development that enabled them to work more independently. ‘Yes, it would have uh helped Ah in terms of also preparing you mentally. The fact that you would have had some sort of practice would have assisted you in being focused when you are with an ICU patient and just give you that reassurance that yes I’ve practiced it a bit and recall your memory in terms of how to do h something.’ (P2, M, 38 years old) 3.2. **The participants acknowledged that professional growth could only be obtained if you take responsibility for your own learning.** ‘I feel like I’m a type of person where I would like to have a challenge, So Ummm I get a challenge in an ICU and it preparer’s me, I’m prepared to face anything because we see different, types of challenges. ICU is learning, you’re always eager to learn.’ (P3, F, 23 years old) 3.3. **The participants experienced that the process of learning is different for each individual and should be embraced as such.** ‘I say as the day goes by the more you learn your things the more you face new challenges. is the more you gain confident, in your profession as per se, and then ending up feeling like right know I am ready for practice.’ (P1, F, 23 years old)

*Source:* Olivier, L., 2022, Practice readiness of final-year nursing students in intensive care in a private hospital group, Unpublished Masters dissertation, University of Johannesburg, Johannesburg

ICU, intensive care unit; P, participant; F, female; M, male.

## Discussion

### Prolonged exposure to the intensive care unit clinical learning environment

Prolonged exposure to the ICU environment influenced the student nurse’s perceived readiness for practice as they were exposed to situational learning opportunities (Pryse et al. 2020; Rush et al. 2019). Voluntary compensated overtime shifts resulted in prolonged exposure to the ICU environment. Students allocated to specialised hospitals were more likely to be exposed to the ICU environment because of rotational placement and the opportunity to work overtime. The results indicated no significant difference in practice readiness scores between students allocated to peripheral and specialised hospitals. These results were surprising. Based on Benner’s theory, novice nurses are expected to develop skills and understanding of patient care over time. Benner’s theory further suggests that knowledge and decision-making skills are gained in specific situations in relation to exposure to the CLE. As a result, it was expected that students who worked more overtime shifts in the ICU and were placed in a specialised hospital would report a higher readiness for practice score.

These results could be explained by the prerequisite requirements that all the respondents had 3 years of experience and clinical exposure in a general ward and met the outcomes stipulated in the curriculum, as described by the *Nursing Act* (*No. 33 of 2005*) prior to their enrolment as a bridging course student. Based on the practice readiness scores and in accordance with Benner’s classification of development, with consideration of prior exposure to the ICU, the newly qualified nurses in this study were classified as novices or, at the most, advanced beginners (Alligood 2014; Benner 2001; Landers et al. 2020; Murray et al. 2019; Thomas & Kellgren 2017).

### Support professional socialisation and belonging

Benner (2001) identifies the need for guidance from preceptors and experts for the novice and advanced beginner as a lifeline in a new and unfamiliar situation. As indicated in Table 7 and confirmed through data triangulation as indicated under Domain 2 and illustrated in Sub-domain 2.1., respondents were mainly supported by the shift leader and their peers. Because of the guidance, supervision and support offered by the shift leader, the students were able to build a trusting relationship that made them feel safe and accepted as valuable members of the team (Murray et al. 2019; Nyiringango et al. 2021). If the shift leader embraced the opportunity to allow the students to benefit from valuable situational learning opportunities, they would deliberately involve the students in these learning moments (Harrison et al. 2020; Wiredu & Roberts 2020). Positive mentors are irreplaceable in facilitating the novice’s transition into a new working environment and promoting professional growth and independence (Comparcini et al. 2020; Nyiringango et al. 2021; Wong & Bressington 2021). The students clearly indicated that the ENA was especially valuable with regard to orientation to the layout, stock placement and routine of the unit as indicated in Domain 2 (Sub-domain 2.1). This was a surprising result as the ENA is considered a junior staff member and the importance of their role in the team dynamics is often overlooked.

### Orientation and skill development

As explained by Benner’s theory, the novice relies on standardised policies and procedures, as well as a set routine, to provide them with structure and a sense of security that they are doing what is expected of them (Pryse et al. 2020; Rush et al. 2019;Song & McCreary 2020). Receiving orientation on the routine in the unit will assist them with prioritising tasks (Alligood 2014; Benner 2001; Landers et al. 2020; Murray et al. 2019; Thomas & Kellgren 2017). Orientation underpins the understanding of what is expected of them as members of the multi-disciplinary team (Rodríguez-García et al. 2018). In the absence of proper orientation, the newly employed nurse will feel overwhelmed, frustrated and incompetent (Pleshkan & Hussey 2020). As per Table 8, the results indicated that the students did not view orientation as one of the three most important factors that influenced their self-perceived readiness for practice in the ICU. However, during the qualitative phase of the study, the participants explained the importance of orientation and how not being orientated affect their ability to function independently as valuable members of the team. The quantitative results could be explained as students were exposed to the ICU during their studies prior to enrolment as bridging course students. Based on prior exposure, they did not feel completely lost or disorientated and were familiar with some of the policies, procedures and routines related to the ICU CLE. Through triangulation of the results, the importance of systematic orientation cannot be ignored as a contributing factor to the nurse’s perceived readiness for practice.

**TABLE 8:** Indicators for readiness for practice.

Variables	β†	95% CI for β	*p*
Quality of orientation	−0.206	−0.248; 0.007	0.064
Support structure general nurse	0.360	0.110; 0.433	0.001
Shifts of overtime worked per month	0.408	0.091; 0.267	< 0.001
Attended up-skill training	−0.208	−1.094; −0.035	0.037

*Source:* Olivier, L. 2022, Practice Readiness Of Final-Year Nursing Students In Intensive Care In A Private Hospital Group, University of Johannesburg, Johannesburg. (Unpublished Masters dissertation)

† , Standardised beta coefficient; CI, confidence interval.

### Skill development through simulation

As indicated in Table 7, students indicated that even though they were given the opportunity to practice some skills in simulation during their basic training, they did not feel competent to perform these skills in the clinical environment. As indicated in Domain 3, Sub-domain 3.1, participants indicated that if they had the opportunity to attend an up-skill programme, they would have been better prepared for practice. Through an up-skill programme, students could have had the opportunity to integrate theory and practice (Jørgensen, Larsen & Gram 2018; Menard & Maas 2019; Tjoflåt, Koyo & Bø 2021). They acknowledged the need to practice the required new skills in a safe environment before being expected to do so in reality (Jørgensen et al. 2018; Menard & Maas 2019; Tjoflåt et al. 2021).

### Recommendations

Multifaceted, innovative introduction programmes may help close the gap, assisting the student to become a novice general nurse who is practice ready. Nurse managers must support and encourage a culture of caring for students, lifelong learning and provision of learning support to students allocated to their units during WIL (Comparcini et al. 2020; Hattingh 2019). This positive culture towards students will foster evidence-based practices and enhance patient safety (Comparcini et al. 2020; Hampton et al. 2021; Harrison et al. 2020). Through the revision and implementation of standardised policies and procedures, the necessary objective guidance and support are provided to students to facilitate the provision of evidence-based safe patient care (Murray et al. 2019; Pitts & Christenbery 2019).

Formally planned, structured unit-specific orientation programmes should be introduced prior to students being allocated to the unit for WIL opportunities (Hampton et al. 2021; Hattingh 2019; Innis & Calleja 2018; Pleshkan & Hussey 2020). Orientation programmes should include an introduction to the unit manager and co-workers, unit routines and teamwork, communication structures, as well as clear expectations regarding team assistance and equipment operation (Hampton et al. 2021; Harrison et al. 2020; Hattingh 2019; Rush et al. 2019; Wiredu & Roberts 2020). Orientation of students should be allocated to a dedicated staff member (Comparcini et al. 2020; Gómez-Ibáñez et al. 2020; Vuckovic et al. 2019).

The appointment of a CNS with a clearly defined role description related to student allocation, facilitation, support, orientation, staff development and training in collaboration with the clinical facilitator will contribute to a positive culture that promotes lifelong learning (Comparcini et al. 2020; Hattingh 2019). Additionally, the implementation of a mentor system, where students can engage in professional socialisation that promotes personal and professional growth, will promote a feeling of belonging (Comparcini et al. 2020; Hampton et al. 2021; Pleshkan & Hussey 2020). To enhance peer support, the allocation of at least two students to the same ICU on the same shift is recommended (Price et al. 2018; Rush et al. 2019; Vuckovic et al. 2019).

All final-year students should be afforded the opportunity to attend an upskilling programme prior to the commencement of allocation to an ICU. Such programmes will ensure competence in performing the basic skills required to participate as a functional member of the team (Deschênes et al. 2019; Lee, Kim & Chae 2020; Sterner et al. 2019). Skills should be practised in a simulation until competence is demonstrated and reinforced during WIL under the direct supervision of the clinical nurse specialist (CNS), shift leader (SL) and mentor (Deschênes et al. 2019; Lee et al. 2020; Sterner et al. 2019).

The PEI should continuously monitor and improve implemented transition programmes through trial and error. These transition programmes should be shared, standardised and implemented on a national level within the PEI and their affiliated healthcare providers to promote the accessibility of a well-developed transition programme to all students (Hattingh 2019).

### Strengths and limitations

The study’s results are limited to students registered for the bridging course (R683) and should not be generalised. The sample population for the *t*-test based on the study’s hypothesis consisted of only 44 respondents. Most respondents (34) were allocated to specialised hospitals, and it is thus possible that the results could be biased. This study was sequential and explanatory and employed a mixed-method approach. The results obtained during the first phase were confirmed and elaborated on during the study’s second phase. It appears to be the first study in South Africa that explored the possibility of placing newly qualified general nurses in the ICU setting. Valuable insights were gained from this mixed-method approach, and the recommendations were aimed at developing support programmes. These support programmes could bridge the gap between being a student and becoming a novice general nurse who could transition into the ICU. These recommendations could promote a smooth transition into practice for all categories of future nursing students and newly employed staff in the ICU. By implementing the recommendations, staff retention in the ICU and burnout could be positively influenced.

## Conclusion

The preparation-practice gap is a reality and a threat to quality patient care (Innis & Calleja 2018). To enable the novice to provide quality patient care in the intensive care setting, adequate objective and subjective support that contributes to the novice’s sense of security needs to be prioritised (Comparcini et al. 2020; Hampton et al. 2021; Harrison et al. 2020). The development of professional competence and the ability to take responsibility can only be obtained through experience-based knowledge supported by continuous professional development programmes (Hampton et al. 2021; Innes & Calleja 2018; Splitgerber et al. 2021). The quality of patient care can be improved by the development and implementation of transitional programmes aimed at developing the novice’s ability to accept responsibility and demonstrate accountability, by self-recognition of their limitations, exercising professional judgement and seeking consultation with their seniors and role models (Cheraghi et al. 2021; Gómez-Ibáñez et al. 2020; Innes & Calleja 2018; Murray et al. 2019). As each learning environment is unique and context specific, it is important to identify the factors that are predictive of readiness for each situation (Casey et al. 2021). Only once these factors are identified and understood within the context can transition programmes be developed to promote practice readiness (Casey et al. 2021).
